# Addressing ethnic disparity in antenatal care: a qualitative evaluation of midwives’ experiences with the MAMAACT intervention

**DOI:** 10.1186/s12884-020-2807-4

**Published:** 2020-02-19

**Authors:** Helle Johnsen, Nazila Ghavami Kivi, Cecilie H. Morrison, Mette Juhl, Ulla Christensen, Sarah F. Villadsen

**Affiliations:** 1 Department of Midwifery and Therapeutic Sciences, University College Copenhagen, Sigurdsgade 26, 2200 Copenhagen N, Denmark; 2 Section of Social Medicine, Department of Public Health, University of Copenhagen, Øster Farimagsgade 5, Postboks 2099, 1014 Copenhagen K, Denmark; 3 Danish Institute for Study Abroad, Vestergade 7, 1456 Copenhagen, Denmark; 4 Section of Women’s diseases, Pregnancy and Childbirth, Herlev Hospital, Borgmester Ib Juuls Vej 1, 2730 Herlev, Denmark

**Keywords:** Maternal and child health, Antenatal care, Pregnancy, Complex interventions, Inequity, Ethnicity, Migration

## Abstract

**Background:**

In Denmark, 13% of all children are born by non-Western immigrant women. The public antenatal care has not adapted to this increased diversity of women. Compared to women coming from Western countries, non-Western immigrant women have an increased prevalence of severe maternal morbidity and higher risks of maternal death, stillbirth and infant death. Suboptimal care is a contributing factor to these ethnic disparities, and thus the provision of appropriate antenatal care services is pivotal to reducing these disparities and challenges to public health. Yet, little is known about the targeted interventions which have been developed to reduce these inequities in reproductive health. The MAMAACT intervention, which included a training course for midwives, a leaflet and a mobile application, as well as additional visit time, was developed and tested at a maternity ward to increase responses to pregnancy warning signs among midwives and non-Western immigrant women. Aim: To explore the feasibility and acceptability of the MAMAACT intervention among midwives and identify factors affecting midwives’ delivery of the intervention.

**Methods:**

Eight mini-group interviews with midwives (*n* = 18) were undertaken. Systematic text condensation was used to analyse data.

**Results:**

Three main categories were identified, which were ‘Challenges of working with non-Western immigrant women’, ‘Attitudes towards and use of the leaflet and mobile application’, and ‘Organisational factors affecting the use of the MAMAACT intervention’.

**Conclusions:**

The MAMAACT intervention was found to be feasible as well as acceptable among midwives. Women turning to relatives for pregnancy-related advice, time constraints during midwifery visits, incomplete clinical records and lack of professional interpreter assistance impacted midwives’ delivery of the MAMAACT intervention. Midwives displayed a readiness for the MAMAACT intervention; however, there is a need to further examine how contextual factors may impact the use of the intervention in antenatal care.

**Trial registration:**

ClinicalTrials.gov, Retrospective Registration (07/2/2020), registration number NCT04261400.

## Background

Immigration to Denmark has increased significantly in recent years [1]. Currently, 13% of children are born by non-Western immigrant mothers [1]. Antenatal care is publicly funded and free of charge for women with residence in Denmark [2]. For women with uncomplicated pregnancies, the antenatal care program includes approximately five visits to the midwife, three visits to the general practitioner and two ultrasound examinations [2]. Despite access to free antenatal care in Denmark [2], immigrant women have lower antenatal care utilisation [2, 3].

In Europe, studies point to immigrant women having a higher risk of negative pregnancy and birth outcomes compared to the native populations [4, 5]. Studies have shown that, during pregnancy, some groups of immigrant women have an elevated risk of severe maternal morbidity compared to women born in high-income countries [6–8]. The direction and strength of the risk vary depending on the immigrant’s country of origin, the specific outcome and the new national setting [4–6, 9]. In Denmark, ethnic inequities in stillbirth, infant and child mortality have been found in offspring to immigrant women born in Turkey, Pakistan and Somalia [10]. Poor health status at birth can impair the cognitive, sensory and motor development, and lead to learning disabilities [11], thus reducing both the potential for a long and healthy life for the individual and the equality in life chances. The mechanisms behind poorer maternity outcomes in immigrant groups are complex and should be understood in a life course perspective including elements from before the migration, the migration process itself and the resettlement in a new country [5, 12]. In the new country, maternal health is often affected by low socioeconomic position [6], low health literacy levels, and chronic stress [5]. In addition, it has consistently been shown that immigrant women are more likely to receive suboptimal maternity care [4, 5, 8, 13, 14]. Miscommunication, language barriers, delays in care-seeking and lack of adherence to clinical guidelines are among the main explanations for these results. Similar tendencies of suboptimal care have been found in Denmark, where non-Western immigrants were more likely to experience the death of a child during birth [15], which is a well-known indicator for the quality of care [16]. These findings highlight the need to improve the response to pregnancy complications among immigrant women in western countries.

The World Health Organization recommends the improvement of health education materials on signs of pregnancy complications and health system navigation in women’s native languages, as well as adopting a person-centered, diversity-sensitive model of care [4]. Yet, more detailed guidance on how to comply with this recommendation is lacking [4]. Work is being conducted to develop means to improve the cultural competence, as well as the cultural awareness and sensitivity, of health care providers and points to the training of health care providers as a useful tool [17]. However, within the field of maternity care in the European region, there has to our knowledge been no scientific studies of this type of initiative, including how maternity care providers respond to initiatives aiming to change their communication strategies [4, 17]. This article reports on the evaluation of the MAMAACT intervention, which was developed to promote the response to warning signs of pregnancy complications among non-Western immigrant women and midwives. The training of midwives in cultural competence and increased attention to counselling on signs of pregnancy complications and health system navigation were hypothesised to improve the management of pregnancy complications. In complex interventions, diverse forms of evaluation evidence are needed to inform decision making [18, 19]. Qualitative research can contribute with insights into how stakeholders accept an intervention and this is useful for considerations of its potential and transferability [20].

## Methods

### Aim

The aim of this study was to explore the feasibility and acceptability of the MAMAACT intervention among midwives and identify factors affecting midwives’ delivery of the intervention.

### The MAMAACT intervention

The MAMAACT project was initially a subproject under “Towards Sustainable Healthy Lifestyles Interventions for Migrants” [3]. The project’s aim was to reduce ethnic disparity in stillbirth and infant death by improving the management of pregnancy complications through timely and appropriate response to warning signs of pregnancy [21]. To assure that intervention development met immigrant women’s needs within the Danish antenatal care context, a mixed-methods needs assessment was performed. A register study mapped immigrant women’s antenatal care utilisation patterns and a case series-study described and analysed causes and characteristics of perinatal deaths according to the maternal country of origin at Denmark’s largest maternity ward [3, 15, 21]. In addition, interviews and observations including non-Western immigrant women, midwives, general practitioners and community nurses addressed target group and maternity care providers’ needs as well as the organisational possibilities for implementing an intervention in antenatal care [3, 21]. Findings from these studies were used to guide the subsequent intervention development.

**Fig. 1:**
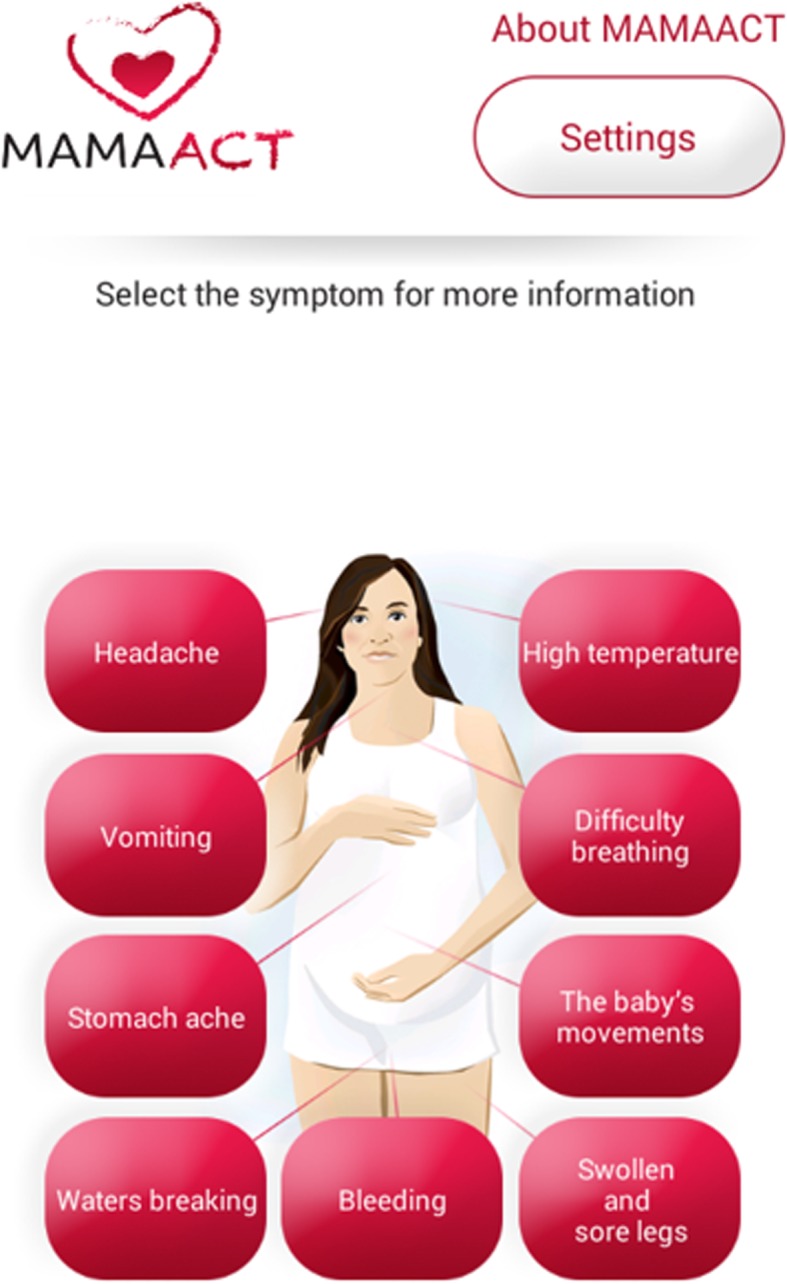
The MAMAACT app

The MAMAACT intervention was developed as a complex intervention [18] in cooperation with midwives at Denmark’s largest maternity ward. The intervention comprised of the following components: a 5-h training session for midwives in cultural competence [22] followed by three dialogue meetings, a leaflet and a mobile application (app) describing the response to warning signs during pregnancy for women, and the possibility to extend the first midwifery visit by 5 min [21]. The training content was developed by operationalising the concept of cultural competence, including knowledge, awareness and skills, among the midwives [22]. During the training session, midwives were introduced to the intervention’s empirical background. They worked with ‘best practice’ for care provision in highly diverse settings with specialists from the Migrant Medical Clinic at the hospital and with audit-inspired cases based on recent perinatal deaths. Finally, they were taught about the different elements in the leaflet and app. To improve the adaptation of the intervention to the local antenatal facility context [18], midwives were encouraged to introduce and follow up on the MAMAACT material as this was found to be most appropriate for the individual woman and the specific visit. The dialogue meetings functioned as a tool to refresh learning from the training course and sought to share experiences and promote reflection on intervention activities among the midwives. The leaflet contained written information about common pregnancy complication symptoms and how to respond to them. The app contained information on the same symptoms as the leaflet in a more elaborated version. As the educational level among non-Western immigrants in Denmark was significantly lower than among the host population [23], the information was phrased in simple language to improve its readability. To complement the written information, anatomical illustrations accompanied the text segments. In addition, the app was fitted with an audio function for women with illiteracy or low levels of literacy [21] (Fig. 1). Both the leaflet and app were translated into Arabic, Persian, English, Somali, Turkish and Urdu, as these non-Western languages were the most predominant in Denmark at the time of the study [23].

Following recommendations from The Medical Research Council [18], the MAMAACT intervention was tested at two antenatal care facilities from 2014 to 2015 prior to planning a nationwide implementation. One of the antenatal care facilities was located in an urban setting, while the other was located in a provincial setting. They served a high level of ethnically diverse populations, as well as a large proportion of low and middle-income households. The MAMAACT leaflet and app were distributed to all pregnant women, equivalent *n* = 2000, attending antenatal care during the test period [3, 21]. Evaluation data consisted of questionnaires for women before and after the intervention, plus focus group interviews with midwives [3]. The questionnaire invitation was distributed to 1790 women; however, due to a low response rate (28%/29%) [3], questionnaire results were not published.

### Data and participants

When investigating aspects related to acceptability and feasibility, a qualitative study design is recommended [24, 25]. In this study, the primary data source is eight semi-structured mini-group interviews with midwives (*n* = 18). In addition, summaries from all dialogue meetings (*n* = 13) allowed for further perspectives and contributed to the development of an interview guide. Dialogue meetings and interviews were undertaken by authors Nazila Ghavami Kivi (NGK) and Cecilie Hjorth Morrison (CHM) from April 2014 to November 2014. Purposeful sampling [26] was used to recruit midwives from the two antenatal care facilities in the study. Inclusion criteria were performing midwifery visits at one of the antenatal care facilities during the intervention test period. Midwives were recruited by the local management staff. They were all female, they had varying degrees of professional experience ranging from less than 1 year to 14 years, and their ages ranged from 25 to 46 years (average 33 years).

### Data collection

To ensure adequate time for sharing professional experiences and increase trust among participants, mini-groups were chosen [27]. A semi-structured interview guide was used to collect data. The interview guide was pilot tested among five midwives. Minor alterations were performed to the guide following the pilot test. In the final interview guide, the main questions were centred around midwives’ perceptions of the MAMAACT intervention, how the intervention was used, communication about pregnancy symptoms and factors affecting care provision. The average interview duration was 1 h. The interviews took place at the antenatal care facility. Midwives were allocated work time to participate. All dialogue meetings and interviews were audio-recorded, and interviews were subsequently transcribed verbatim.

### Data analysis

Data were analysed using systematic text condensation [26]. This method consists of four analytical steps [26]. During step one, ‘total impression’, data was read and reread to gain an overview and identify preliminary themes. In step two, ‘identifying and sorting meaning units’, meaning units were selected and sorted into code groups. Step three, ‘condensation of units and themes’, involved reducing data and decontextualising meaning units by sorting data as thematic codes across the study participants. In step four, ‘synthesising’, results from step three were synthesised. Authors Helle Johnsen (HJ) and Sarah Fredsted Villadsen (SFV) undertook analytical steps one and two. HJ undertook analytical step three. During step four, the analysis was discussed among all authors to ensure that the final categories and sub-categories were grounded in the midwives’ narratives and covered the dataset as a whole.

### Ethical considerations

Midwives received written and verbal information about the study before verbally consenting to participate. Furthermore, they were guaranteed personal anonymity. The names of midwives presented in the following results section are fictitious.

## Results

During the data analysis, three main categories, each with two sub-categories, emerged. The three main categories were ‘Challenges of working with non-Western immigrant women’, ‘Attitudes towards and use of the leaflet and app’, and ‘Organisational factors affecting the use of the MAMAACT intervention’.

### Challenges of working with non-Western immigrant women

#### Passivity and lack of knowledge

Midwives described many non-Western immigrant women as having had experiences with more authoritative healthcare systems and, thus, these women were not used to taking an active role during the midwifery visits. Midwives found that women who were better educated and proficient in Danish came prepared and were more explicit about their needs were easier to communicate with. Some immigrant women were found to be difficult to communicate with. Midwives also explained that a lack of experience with the Danish antenatal care system, could lead to doubts about options and midwifery services:

”*…One (woman) came with a really bad knee ache…the only thing she used the interpreter for was to say that her knee was hurting…I couldn’t help her with that*.”

(Katrine, F6)

Many non-Western immigrant women were described as being less likely to search for pregnancy-related information or to participate in antenatal classes. Instead, midwives reported that immigrant women mainly drew upon their families for advice. Some midwives felt that relatives provided inadequate or incorrect advice and that relatives complicated mutual trust and dialogue between the woman and the midwife.

Some midwives reported a lack of physiological knowledge among non-Western immigrants. Midwives perceived human reproduction to be taboo in certain countries and cultures and women were described to lack knowledge about anatomy, for example, knowing what a uterus was. Midwives felt that this lack of knowledge could lead to decreased physical awareness and less use of intuition causing an inadequate response to pregnancy symptoms:

”*…They don’t have that kind of knowledge about their body, their lower body…and that’s why they don’t react to things happening…we can’t give them an anatomy course.*”

(Marianne, F5)

#### Responses to women’s body symptoms

Many midwives described non-Western immigrant women as presenting more diffuse physical symptoms. They often experienced several symptoms simultaneously. Some midwives described non-Western immigrant women to have a lower threshold for expressing discomforts during pregnancy compared to Danish-born women. Being affected by psychosocial problems and stating numerous physical symptoms were seen as a way of expressing difficult life circumstances in general. Consequently, some midwives saw pain tolerance as affected by ethnicity:

”*…Young ethnic women…they don’t talk about it at home…that it’s okay to have pain. I just saw a woman who had pains all over her body... yes, you have aches, you breathe for two, you are out of breath, that’s normal…*”

(Maria, F7)

Although some midwives were likely to categorise non-Western immigrant women by their country of origin, culture and ethnicity, they also found that they shared challenges with disadvantaged groups of Danish-born women and that socioeconomic status impacted non-Western women’s behaviour more than their ethnicity. Data suggested that, after participating in the MAMAACT training session, many midwives reflected more on their perceptions of women as well as how categorisations could contribute to differential treatment between ethnic Danish women and non-Western immigrant women:

”*…They (their symptoms) are more confusing, we get tired of listening to them…they are not heard as easily as women who are more educated…and who knows how we (Danish people) talk to a doctor…*”

(Tina, F8)

### Attitudes towards and use of the leaflet and app

#### Something tangible to take home

In general, midwives were very positive towards the MAMAACT leaflet and app. The leaflet was found to be easy to read. According to the midwives, the leaflet and app were also well received by women and their partners. The app was considered to be beneficial because it could be accessed via a smartphone.

General information on the organisation of and access to services at the local maternity ward was posted online. Thus, the MAMAACT leaflet was the only material being distributed to pregnant women. Online information was mainly in Danish and having the leaflet and app in six different languages was considered an advantage. Newly arrived immigrant women were described as having greater difficulties navigating antenatal care. Not knowing who to call was a common challenge. Several midwives mentioned the advantage of giving women something to take home, hoping that this would contribute to an increased reaction to pregnancy symptoms:

*“Take the midwife home with you…we can’t knock on the door at seven o’clock at night and ask if she felt (the baby move)…you remind them when you are not sitting beside them…*”

(Janne, F1)

#### Introducing the leaflet and following up

Although midwives generally found the training course relevant, they also felt that the introduction on how to use the MAMAACT material was inadequate:

”… *At the introductory meeting (the training session)… something more practical was missing…how are we expected to communicate it (the MAMAACT material)?…this part is really important…*” (Julie, F5)

Some midwives introduced the leaflet and app as part of a research project or as an option the women could choose. Others introduced the leaflet information and actively encouraged the women to download the app. When women had a non-Western background and were at risk, midwives expressed using more time to introduce the MAMAACT material. Some midwives assessed the material to be less relevant and potentially inducing unnecessary concerns to women with expected normal pregnancies or to psychologically vulnerable women. These reservations were more pronounced at the beginning of the test period. With more experience of working with the material, midwives described finding different communication strategies and adapting information to the woman’s level of anxiety.

Although all midwives introduced the MAMAACT leaflet and app at the first midwifery visit, almost none of them followed up on this during subsequent visits. Midwives assumed that women were using the leaflet and app. Some midwives had heard women referring to ‘the leaflet’ on the delivery ward, but they were uncertain if these women meant the MAMAACT leaflet. Reasons for lack of follow up were forgetting to do so and time constraints:

“*We have several other tasks we have to do at the 28*^*th-*^ *week midwifery visit*.” (Tina, F8)

### Organisational factors affecting the use of the MAMAACT intervention

#### Timeframe and attendance

One component of the MAMAACT intervention was to extend the first midwifery visit by 5 min. However, due to difficulties with the electronic booking system, the first midwifery visit was in fact not extended. This resulted in frustration among a few of the midwives because they found that introducing the leaflet and app to non-Western immigrant women took time. Most midwives felt that they should talk about pregnancy symptoms in any case, and thus implementing the intervention within the existing timeframe was considered to be feasible.

Nonetheless, time was reported as being very important for communicating with women about warning signs during pregnancy. Non-Western immigrant women were described to be likely to arrive late for their visit. The antenatal care schedule did not allow for time flexibility, and hence midwives found it difficult to provide adequate care when schedules were delayed. Furthermore, visits with non-Western immigrant women generally took longer due to communication difficulties. In addition, clinical records for immigrant women could sometimes be incomplete. Lack of information in the clinical record affected midwives’ opportunity to assess women’s care needs and increased the risk of delays in referrals to specialist care. Some midwives had experiences of unintended episodes caused by these clinical record insufficiencies, as described in the following example:

“…*She had been seen in week 17 and then she didn’t come back until week 37 due to a mistake with her booking …she didn’t know whom to call and she didn’t speak Danish…*” (Anne, F2)

#### Language proficiency

Language proficiency was of great importance for the provision of care. Midwives had concerns about communication difficulties causing adverse events. Many non-Western immigrant women were described as lacking the ability to express themselves in Danish or English. Even though the hospital offered interpreter assistance, interpreters were not always available for the midwifery visits. Sometimes, immigrant women would bring their partner, a relative or a friend to interpret for them. This was described as potentially problematic due to the lack of confidentiality and the ability to assess the quality of the translation. Midwives could be uncertain if women’s symptoms were described accurately and if their information and advice were conveyed as intended. In situations where no interpreters or family members were available to interpret, midwives would try to get by using gestures or simple words to assess the health of the mother and the baby:

“*…Baby okay, baby not okay?... you can communicate about necessities, but you can’t have a nuanced conversation*.” (Marianne, F5)

## Discussion

So far, little is known about how interventions may be successful in addressing ethnic disparities in maternity care [4]. Within Europe, immigrant targeted initiatives include group-based antenatal care and doula support [28–31]. To our knowledge, the MAMAACT intervention is the first complex intervention developed specifically to increase response to pregnancy complications among midwives and non-Western immigrant women. Our findings contribute with new insights into how midwives as key stakeholders may influence the implementation of an immigrant-targeted intervention and the importance of a supportive organisational environment for the success and sustainability of such an intervention.

The analysis revealed that despite attending the MAMAACT training course in cultural competence, some midwives were likely to categorise and tended to stereotype non-Western women. Similar findings have been presented in other studies, showing that maternity care providers use ethnicity and cultural beliefs to explain behaviour among immigrant women [32, 33]. In this study, some midwives found non-Western immigrant women to have lower pain tolerance, compared to ethnic Danish Women. Tait and Chibnall assert that provider stereotypes concerning race and ethnicity as well as the circumstances in which the provider-patient interaction takes place are both liable to impact clinical judgments [34]. Thus, midwives’ perceptions of non-Western immigrant women may have impacted how these midwives assessed and responded to the women’s symptoms. In addition, difficult circumstances caused by time restrictions and task loads are likely to have influenced midwives’ communication strategies.

Interestingly, midwives also expressed how they had become more aware of how they interacted with non-Western immigrant women. These findings suggest the training course followed by the dialogue meetings were successful to some extent in promoting a change of action among midwives. The training session for midwives was an operationalisation of the concept of cultural competence [22], which is considered to be a generic competence of extra importance when cultural, ethnic and social differences between the health providers and women attending care are significant [22, 35]. In line with this, the MAMAACT intervention was implemented as a universal intervention. Recognising that this competence is a reflective practice [22], the dialogue meetings were short follow-up sessions for continued inter-colleague thinking and sharing of experience. Previously, the term cultural competence has been misused with a static understanding of culture [36]. In a recent review of health workforce cultural competency interventions, such an interpretation of culture was considered as categorical cultural competence and it was criticised for potentially increasing cultural misunderstandings [37]. Nonetheless, cross-cultural approaches to cultural competence interventions were identified as having a focus on training general knowledge, attitudes and skills that are relevant to navigate in cross-cultural interactions [37]. Furthermore, the cross-cultural approach was found to have positive effects on the attitudes, knowledge and skills of healthcare providers, indicating that using the cultural competence framework in the training of midwives may have been a suitable approach. However, for stronger evidence on the value of cultural competence interventions, there is a need for further development of methods that can be used to measure the effect of these interventions on healthcare provision as well as health outcomes [37, 38].

Overall, the midwives found the MAMAACT intervention to be very relevant, indicating that the leaflet and app were an acceptable approach to attempt to increase response to pregnancy symptoms. However, while midwives found the usability of the leaflet and app to be high, they found the training course to lack information on how to communicate the MAMAACT material. These findings suggest that training in cultural competence may need to be supplemented with more practical communication tools. Although the midwives introduced the leaflet and app at the first midwifery visit, almost none of the midwives followed up on the use of the material during the following visits due to competing tasks or forgetting to do so. Lack of follow up affected the overall compliance with the intervention. In addition, while most midwives used the MAMAACT material to guide their communication, a few midwives introduced this material as a research project, indicating that training in how to introduce the MAMAACT material may have been needed instead of allowing midwives the flexibility to introduce the material as they preferred. This may also have negatively impacted women’s motivation to use the MAMACT intervention, as an incentive to participate in research can be driven by perceptions of personal relevance and gain [39]. Finally, difficulties with the electronic booking system meant that an extension of the first midwifery visit was not possible. Nevertheless, all participating midwives introduced the MAMAACT material during the first visit, suggesting that the intervention was largely feasible under real-world conditions [40] at the local antenatal care facilities included in the study.

A few midwives felt that the MAMAACT material focused solely on pregnancy risks leading to a more biomedical model of antenatal care. This may be due to the fact that, in Denmark, midwives are trained to provide woman-centered care in antenatal care [41, 42], a care model which emphasises emotional and social support in addition to medical care. When work routines change, midwives as stakeholders will use mental models in the form of existing logic to make sense of how the world is different from the expected state of the world [43, 44]. Mental models determine individual perceptions and appraisals of an intervention, and thus they will be a deciding factor in how midwives react to different intervention activities [43]. These midwives’ concerns demonstrate how their mental models may have affected their readiness for change.

Midwives experienced several barriers related to the organisation of antenatal care visits. Lack of professional interpreter assistance affected communication. Previous studies have shown that professional interpreter assistance is pivotal to attaining detailed descriptions from patients, as well as providing adequate advice [32, 45]. Furthermore, midwives found non-Western immigrant women to be a challenging group of women to provide care for. Women arriving late for their midwifery visits, combined with the lack of schedule flexibility affected midwives’ time to perform work routines. Another challenge was that some non-Western immigrant women appeared to prefer pregnancy-related advice from their relatives rather than the midwife. These factors are all likely to have affected midwives’ ability to deliver the MAMAACT intervention [19].

This study has strengths and limitations. One strength is the use of investigator and analyst triangulation [46], as data were collected and coded by two authors and sub and head categories were extensively discussed among all authors. This increases the reliability of the study findings [27]. One limitation is the size of the study, as it only included 18 midwives. However, using material from the dialogue meetings did validate our analysis and contributed to the analytical reflections. Studies investigating intervention feasibility are highly dependent on the context in which they take place [47]. However, in this study, the MAMAACT intervention was tested at two different antenatal care facilities located in areas with high ethnic diversity and low-income households. Furthermore, the clinical settings at these facilities were not protected from the pressures of typical antenatal care provision. This may contribute to the applicability of study findings to other antenatal care settings [40].

## Conclusions

Overall, the MAMAACT intervention was found to be feasible as well as acceptable among midwives. Women turning to relatives for pregnancy-related advice, time constraints during the midwifery visit, incomplete clinical records and lack of professional interpreter assistance all impacted midwives’ delivery of the MAMAACT intervention. Findings from this study suggest that midwives were ready to address problems relating to the provision of antenatal care for non-Western immigrant women. Findings also highlight the need for further analysis of the organisational context surrounding midwives’ efforts to reduce ethnic inequity in reproductive health. In addition, there is a need to include non-Western immigrant women’s experiences with the MAMAACT intervention and their interaction with midwives in antenatal care.

## Data Availability

The datasets analysed during the current study are not publicly available as midwives were not asked to give consent for the transcripts to be published in their entirety. Datasets are available from the corresponding author on reasonable request. Midwives did consent to their professional status, age and number of years as a professional to be disclosed. Quotes in this paper have been selected in a manner which assures the individual source is not identifiable.

## Abbreviations

App: Mobile application
CHM: Cecilie Hjorth Morrison
HJ: Helle Johnsen
MJ: Mette Juhl
NGK: Nazila Ghavami Kivi
SFV: Sarah Fredsted Villadsen
UC: Ulla Christensen
